# Climatic responses and variability in bark anatomical traits of 23 *Picea* species

**DOI:** 10.3389/fpls.2023.1201553

**Published:** 2023-07-17

**Authors:** Wen Nie, Yao Dong, Yifu Liu, Cancan Tan, Ya Wang, Yanchao Yuan, Jianwei Ma, Sanping An, Jianfeng Liu, Wenfa Xiao, Zeping Jiang, Zirui Jia, Junhui Wang

**Affiliations:** ^1^ Key Laboratory of Forest Ecology and Environment of National Forestry and Grassland Administration, Ecology and Nature Conservation Institute, Chinese Academy of Forestry, Beijing, China; ^2^ State Key Laboratory of Tree Genetics and Breeding, Research Institute of Forestry, Chinese Academy of Forestry, Beijing, China; ^3^ Research Institute of Forestry of Xiaolong Mountain, Gansu Provincial Key Laboratory of Secondary Forest Cultivation, Tianshui, China

**Keywords:** bark anatomical traits, climatic response, global distribution, phylogenetic signal, *Picea*

## Abstract

In woody plants, bark is an important protective tissue which can participate in photosynthesis, manage water loss, and transport assimilates. Studying the bark anatomical traits can provide insight into plant environmental adaptation strategies. However, a systematic understanding of the variability in bark anatomical traits and their drivers is lacking in woody plants. In this study, the bark anatomical traits of 23 *Picea* species were determined in a common garden experiment. We analyzed interspecific differences and interpreted the patterns in bark anatomical traits in relation to phylogenetic relationships and climatic factors of each species according to its global distribution. The results showed that there were interspecific differences in bark anatomical traits of *Picea* species. Phloem thickness was positively correlated with parenchyma cell size, possibly related to the roles of parenchyma cells in the radial transport of assimilates. Sieve cell size was negatively correlated with the radial diameter of resin ducts, and differences in sieve cells were possibly related to the formation and expansion of resin ducts. There were no significant phylogenetic signals for any bark anatomical trait, except the tangential diameter of resin ducts. Phloem thickness and parenchyma cell size were affected by temperature-related factors of their native range, while sieve cell size was influenced by precipitation-related factors. Bark anatomical traits were not significantly different under wet and dry climates. This study makes an important contribution to our understanding of variability in bark anatomical traits among *Picea* species and their ecological adaptations.

## 1 Introduction

In woody plants, bark refers to all tissues outside the vascular cambium and is an important component of the stem (Evert, 2006). Structurally, it is divided into two distinct parts: the outer bark (OB) and the inner bark (IB). The OB consists of dead cells, generally the rhytidome, and serves functions such as mechanical support and protection against pathogens. The IB, on the other hand, consists of living tissues including the phloem, and is responsible for the storage and transport of water and photosynthetic assimilates (Rosell et al., 2017). Bark plays important roles in the basic physiological functions of woody plants, and the sizes of tissues and cell morphologies in the periderm and phloem are determined by several developmental pathways and affect plant functions (Srivastava, 1964; Paine et al., 2010; Rosell, 2019). Bark morphology and structure are closely related to the physiological and ecological processes of plants. For example, the cortex in the bark contains chloroplasts capable of photosynthesis, which convert carbon dioxide produced by mitochondrial respiration and flowing in the xylem into sugars (Pfanz, 2008). It also increases bark oxygen concentration and reduces plant stem hypoxia (Wittmann and Pfanz, 2018). Furthermore, the phellem and lenticels in the bark structure regulate the exchange of water, oxygen, and carbon dioxide between the stem and its environment (Lendzian, 2006). For example, the phellem cells have suberin, a waxy substance that makes them impermeable to gases and water. Loram-Lourenço et al. (2022) found that the water conductance of bark across species was related to the morphoanatomical characteristics of the outer bark (i.e., thickness, density, and lenticel investment), while these outer bark characteristics were related to stem transpiration and respiration. For example, the phellem on the bark surface, which consists of dead cells, effectively prevents excessive water loss from the plant (Leite and Pereira, 2017). Therefore, bark anatomical traits are important to clarify their multifunctionality, resource allocation trade-offs, and environmental adaptative mechanisms (Rosell, 2019).

Environmental adaptability is apparent in the ecological strategies of most plant organs and tissues, including bark anatomical traits (Wright et al., 2004; Freschet et al., 2021; Yang et al., 2022). Differences in bark thickness and tissue structure can develop under stress, and such changes are associated with plant resilience (Kopanina et al., 2022). The phylogenetic niche conservatism hypothesis suggests that more closely related species are more likely to have similar functional traits and that conserved and similar functional traits will exhibit stronger phylogenetic signals (Blomberg et al., 2003; Losos, 2008). Plant anatomical traits can exhibit a wide range of variation due to climate-driven effects, for example, xylem vessels of plants in arid regions are often characterized by narrow lumens and thick walls (Baas et al., 1983). Many previous studies have shown that bark structural characteristics are related to fire factors (Lawes et al., 2013; Pausas, 2015) and that species from fire-prone areas tend to have thicker bark that better protects phloem tissues from destruction. Thus, it is important to study how bark anatomical traits vary due to environmental factors in the context of phylogeny.

There are about 40 *Picea* species worldwide, they are widely distributed in boreal, temperate, and subtropical high-altitude regions of the northern hemisphere (Farjon, 2001), making up significant portions of forests. *Picea* species provide ecological benefits and their bark is well utilized as a forest by-product (Harkin, 1971). For example, spruce bark extract is a natural antioxidant and anti-inflammatory agent. According to the information recorded by the Global Biodiversity Information Facility (GBIF, http://www.gbif.org), this genus has been widely introduced to various regions of the world and has strong environmental adaptability. Previous studies on *Picea* species have focused on the morphological and anatomical traits of wood (Piermattei et al., 2020; Puchi et al., 2020), pollen (Jia et al., 2014), and needles (Wang et al., 2021). Importantly, the organ or tissue anatomical traits of *Picea* species have been shown to be closely related to their physiological and ecological functions. For example, certain needle anatomical traits determine photosynthetic performance (Wang et al., 2021), and xylem cell number and cell lumen area affect the hydraulic systems of trees (Sperry and Tyree, 1988; Puchi et al., 2020). The structure and function of the bark influence water transport and storage in the plant. For example, low density bark has a higher hydraulic conductivity (Loram-Lourenço et al., 2020). Indeed, the organ or tissue anatomical traits in *Picea* can provide new insight into interspecific relationships and the mechanisms by which biotic and abiotic factors affect them. However, studies on the interspecific variation in bark anatomical traits and the underlying driving mechanisms in species of this genus are lacking.

In this study, the bark anatomical traits of 23 *Picea* species native to North America, Europe, and Asia were examined in conjunction with information on climatic factors of the *Picea* species. This was done to address the following questions: (1) are there interspecific differences in bark anatomical traits among *Picea* species and are anatomical traits phylogenetically conserved among species in different habitats; (2) are there trade-offs in changes among bark anatomical traits; and (3) what are the climatic factors driving variation in bark anatomical traits of *Picea*?

## 2 Materials and methods

### 2.1 Bark sample collection

The samples were collected from the experimental nursery of outdoor in the Shaba experimental base of the Research Institute of Forestry of Xiaolong Mountain in Gansu Province, China (104°38’E, 34°07’N). The area has an elevation of 1,550–2,100 m, average annual temperature of 7.2°C, average annual precipitation of 757 mm, and average relative humidity of 78%, and the growing conditions are similar for all *Picea* species in the nursery. In October 2020, 23 *Picea* species from the experimental nursery were sampled. All species were sown in 2008. After three years of cultivation, individual trees were transplanted into the nursery with 1.5 m of spacing between them. For each *Picea* species, three single plants of uniform size and normal growth were selected and bark samples 2 × 2 cm in size were taken at 30 cm from the base of each plant. Bark samples include all tissues peeled from the cambium to the surface of the bark.

### 2.2 Bark sample fixation

Referring to the method of fixation in needles and pollen of *Picea* (Jia et al., 2014; Wang et al., 2021), bark sections were prepared using the following 10 steps: (1) Sample fixation: Bark samples were fixed with FAA fixative (90 mL ethanol + 5 mL formaldehyde + 5 mL acetic acid) for 24 h. (2) Sample dehydration waxing: Cut 2–3 mm bark samples along the tangential direction and rinsed in running water for 30 min, placed in 15% ethanol for 2 h, and transferred to a dehydrator (DIAPATH, Donatello) for dehydration. (3) Sample embedding: Melted wax was poured into an embedding frame and before the wax solidified the tissue was removed from the dehydration box and placed into the frame according to the embedding surface. Finally, the samples were cooled on the -20°C freezing table to solidify the wax, which was then trimmed. (4) Sample sectioning: Trimmed wax blocks were placed in a paraffin slicer (Shanghai Leica Instruments Co., Ltd., China, RM2016) and sectioned at a thickness of 4 μm. (5) Sample dewaxing: Sections were dewaxed and hydrated using ethylene glycol monoethyl ether acetate, ethanol solution, and distilled water. (6) Safranin O staining: Sections were placed in safranin O staining solution for 2 min, and then washed briefly in distilled water to remove excess dye. (7) Decolorization: Sections were placed sequentially in a 50%, 70%, and 80% alcohol gradient for 3–8 s each, in order to wash away the excess safranin O staining solution. (8) Fast green staining: Sections were placed in fast green staining solution for 6–20 s and dehydrated in anhydrous ethanol. (9) Sample sealing: Sections were placed in xylene for 5 minutes and sealed with neutral balsam. (10) Microscopic examination: Bark samples were observed using an optical microscope (Nikon Eclipse E100, Japan) and imaged with a high-definition camera (Nikon DS-U3, Japan).

### 2.3 Data acquisition

**Figure 1:**
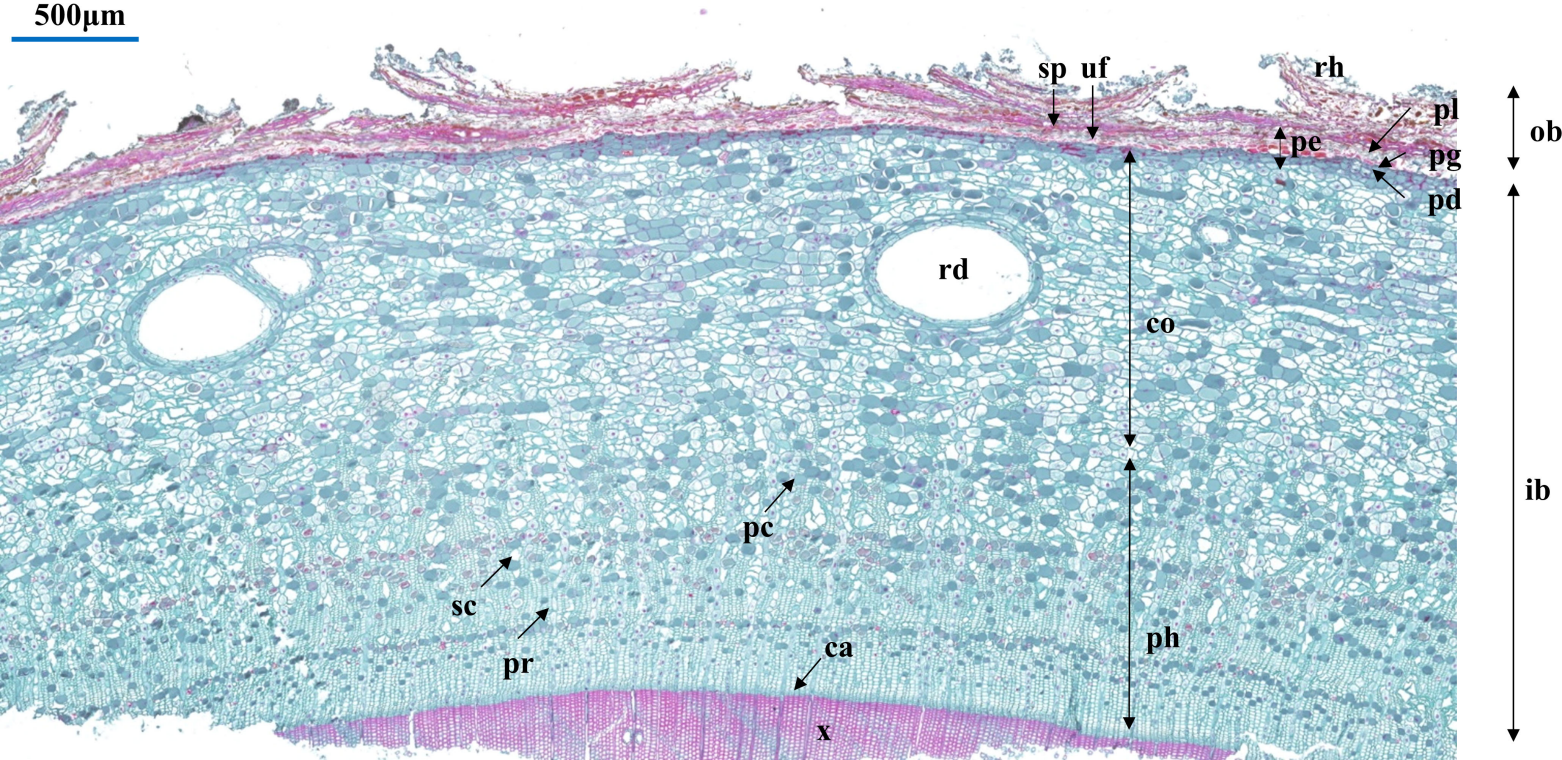
Cross section of bark anatomical traits, exemplified by *Picea engelmannii*. Abbreviations: ca, cambium; co, cortex; ib, inner bark; ob, outer bark; pc, parenchyma cell; pd, phelloderm; pe, periderm; pg, phellogen; ph, phloem; pl, phellem; pr, phloem ray; rd, resin duct; rh, rhytidome; sc, sieve cell; sp, suberized filling tissue or phellem cells with polyphenolic content; uf, unsuberized filling tissue; x, xylem.

We selected three bark samples for each species as biological replicates. Considering the differences between primary and secondary structures in the bark and the difficulty in differentiating them, 30 parenchyma cells and sieve cells were randomly selected from the scanned image of each sample to measure their radial and tangential widths. In this study, bark anatomical sections were measured using the CaseViewer software (3DHISTECH CaseViewer, Budapest, Hungary). The anatomical trait related to bark structure and function were selected for assessment (Angyalossy et al., 2016; Schweingruber et al., 2019; Rosner and Morris, 2022), including (**Table 1**): periderm thickness (PE), cortex thickness (CO), phloem thickness (PH), tangential diameter of resin duct (TR), radial diameter of resin duct (RR), phloem ray width (PR), tangential diameter of sieve cell (TS), radial diameter of sieve cell (RS), tangential diameter of parenchyma cell (TP), and radial diameter of parenchyma cell (RP). In order to reduce the effects of uneven growth of bark cells and tissues, the aspect ratios of some anatomical traits were also calculated, including tangential diameter of resin duct/radial diameter of resin duct (TR/RR), tangential diameter of sieve cell/radial diameter of sieve cell (TS/RS), and tangential diameter of parenchyma cell/radial diameter of parenchyma cell (TP/RP). A detailed bark anatomy is provided in **Figure 1**.

**Table 1:** Bark anatomical traits analyzed.

Abbreviation	Unit	Traits
PE	μm	Periderm thickness
CO	μm	Cortex thickness
PH	μm	Phloem thickness
TR	μm	Tangential diameter of resin duct
RR	μm	Radial diameter of resin duct
PR	μm	Width of phloem ray
TS	μm	Tangential diameter of sieve cell
RS	μm	Radial diameter of sieve cell
TP	μm	Tangential diameter of parenchyma cell
RP	μm	Radial diameter of parenchyma cell
TR/RR	–	Tangential diameter of resin duct/Radial diameter of resin duct
TS/RS	–	Tangential diameter of sieve cell/Radial diameter of sieve cell
TP/RP	–	Tangential diameter of parenchyma cell/Radial diameter of parenchyma cell

**Figure 2:**
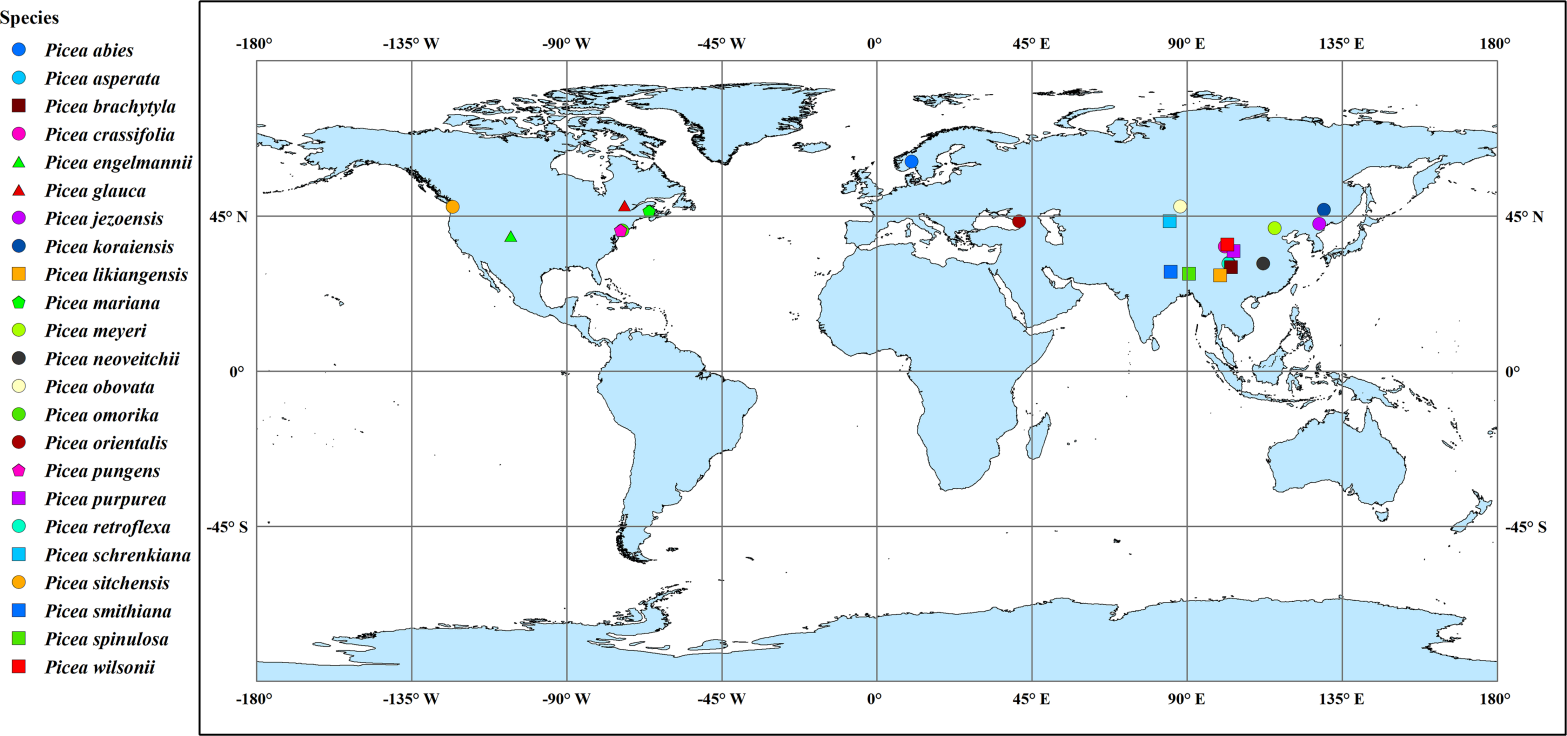
Collection sites of the 23 *Picea* species. For simplicity, the species point distributions use the average latitude and longitude for all source locations. Point shapes and colors distinguish among species.

To further understand the responses and adaptations of bark anatomical traits of *Picea* species to climatic factors, we collected information regarding all original collection sites of the 23 *Picea* species by referencing Ouyang et al. (2021) and GBIF (http://www.gbif.org) (**Figure 2** and **Table 2**). Each species averaged all sites within the GBIF record range to represent the center position of the sampling site. The temperature and precipitation related climate factors were also obtained from WorldClim v2.0 (http://www.worldclim.org) in conjunction with the geographic information of the sources. These included the annual mean temperature, mean temperature of the wettest quarter, mean temperature of the driest quarter, annual precipitation, precipitation of the wettest quarter, and precipitation of the driest quarter. The global aridity index was obtained at the CGIAR consortium for spatial information (CGIAR-CSI, https://cgiarcsi.community) (Zomer et al., 2022). In addition, based on mean annual precipitation, we classified areas with > 500 mm as moist and areas with < 500 mm as dry (Smith et al., 2008). The climate variables for each species were averaged across all source sites for the species using the ‘raster’ package in R 3.6.3 software (R Core Team, 2020).

**Table 2:** Tree size, geographical origin, and climatic information of 23 *Picea* species.

Species	Sources	Ground diameter (cm)	Height (m)	Latitude	Longitude	AMT (°C)	MTWQ (°C)	MTDQ (°C)	APRE (mm)	PREWQ (mm)	PREDQ (mm)	AI
*P. abies*	Norway	9.64 ± 2.26	5.90 ± 0.41	60.77°N	10.09°E	4.86	6.38	2.31	1,039.99	353.92	168.10	1.51
*P. asperata*	Jiulong, Sichuan, China	5.36 ± 0.50	1.83 ± 0.27	31.37°N	102.22°E	7.38	14.56	-1.13	731.31	389.00	15.55	0.72
*P. brachytyla*	Ya’an, Sichuan, China	5.86 ± 0.36	3.32 ± 0.32	30.11°N	102.88°E	12.00	19.10	3.57	973.98	546.71	30.80	0.93
*P. crassifolia*	Datong, Qinghai, China	5.52 ± 1.32	2.02 ± 0.16	36.13°N	101.05°E	1.31	11.27	-9.26	433.06	253.44	6.00	0.41
*P. engelmannii*	Colorado, America	4.09 ± 0.71	1.44 ± 0.26	39.24°N	106.17°W	1.02	7.92	-1.25	571.29	181.16	109.30	0.48
*P. glauca*	Quebec, Canada	6.00 ± 1.67	2.80 ± 0.52	48.06°N	73.13°W	2.37	14.15	-9.49	1,014.93	320.42	183.43	1.29
*P. jezoensis*	Changbai, Jilin, China	4.48 ± 0.44	2.94 ± 0.21	42.69°N	128.42°E	2.99	17.85	-13.72	686.81	423.63	19.94	0.76
*P. koraiensis*	Yichun, Heilongjiang, China	5.34 ± 0.77	2.85 ± 0.15	46.78°N	129.76°E	2.48	19.83	-17.30	601.00	377.00	18.33	0.59
*P. likiangensis*	Diqing, Shangri-La, Yunnan, China	5.56 ± 1.43	2.89 ± 0.61	27.81°N	99.72°E	6.05	12.69	-0.48	703.67	331.67	36.33	0.63
*P. mariana*	New Brunswick, Canada	5.46 ± 2.16	3.08 ± 1.40	46.46°N	66.01°W	4.40	2.96	1.45	1,151.32	325.96	247.95	1.43
*P. meyeri*	Chifeng, Inner Mongolia, China	3.52 ± 0.87	1.89 ± 0.31	41.49°N	115.55°E	5.98	19.95	-10.19	394.00	269.00	9.33	0.29
*P. neoveitchii*	Shennongjia, Hubei, China	6.62 ± 0.65	3.13 ± 0.34	31.20°N	112.16°E	14.45	23.30	3.59	1,122.00	489.50	82.00	0.98
*P. obovata*	Altai, Xinjiang, China	5.06 ± 0.36	1.68 ± 0.31	47.81°N	88.07°E	3.77	19.82	-11.33	183.00	67.00	24.00	0.14
*P. omorika*	New York, USA	4.64 ± 1.22	2.74 ± 0.33	40.86°N	73.88°W	11.88	22.37	0.60	1,174.00	316.00	260.00	0.89
*P. orientalis*	Russia	3.18 ± 0.15	2.22 ± 2.67	43.46°N	41.38°E	3.99	6.46	-3.23	1,276.16	384.42	233.00	1.36
*P. pungens*	New York, USA	6.88 ± 1.34	4.21 ± 0.80	40.90°N	74.34°W	11.64	19.58	0.57	1,148.62	314.20	255.87	0.90
*P. purpurea*	Zhangxian, Gansu, China	5.12 ± 0.24	1.42 ± 0.34	34.81°N	103.64°E	4.56	13.36	-6.15	570.86	304.21	8.50	0.56
*P. retroflexa*	Ma’er Kang, Sichuan, China	5.96 ± 0.37	2.56 ± 0.24	31.25°N	102.06°E	9.88	17.41	1.31	744.88	412.75	15.00	0.66
*P. schrenkiana*	Yili, Xinjiang, China	2.72 ± 0.54	0.93 ± 0.28	43.51°N	85.03°E	7.52	20.67	-8.14	177.72	76.14	19.07	0.13
*P. sitchensis*	Washington, America	7.22 ± 1.32	5.16 ± 0.36	47.66°N	123.01°W	9.78	4.66	15.62	1,897.70	837.38	160.15	2.18
*P. smithiana*	Jilong, Tibet, China	8.40 ± 0.73	5.06 ± 0.36	28.85°N	85.29°E	4.90	-1.35	8.63	407.00	137.00	68.00	0.31
*P. spinulosa*	Rikaze, Tibet, China	4.44 ± 1.07	2.74 ± 0.54	28.30°N	90.62°E	6.28	9.43	2.76	727.00	369.50	36.00	0.63
*P. wilsonii*	Huzhu, Datong, Qinghai, China	5.01 ± 1.39	1.83 ± 0.51	36.75°N	101.82°E	3.05	12.88	-7.46	436.67	255.33	4.00	0.38

AMT, annual mean temperature; MTWQ, mean temperature of the wettest quarter; MTDQ, mean temperature of the driest quarter; APRE, annual precipitation; PREWQ, precipitation of the wettest quarter; PREDQ, precipitation of the driest quarter, AI: aridity index. Ground diameter and tree height were expressed as mean ± standard deviation.

### 2.4 Statistical analysis

The ‘V.PhyloMaker’ package (Jin and Qian, 2019) and the ‘Plantlist’ package (Zhang, 2018) in the R 3.6.3 software were used to elucidate the influence of phylogenetic relationships on *Picea* species attributes. The Pagel’s λ and Blomberg’s K values (Harmon et al., 2008; Kembel et al., 2010) of bark anatomical traits were calculated using phylogenetic trees to assess the phylogenetic signal of bark anatomical traits in *Picea* species. To further elucidate the relationships among bark anatomical traits, phylogenetic independent contrasts (PIC) were calculated for each bark anatomical trait using the ‘ape’ package (Felsenstein, 1985). General Linear Model (GLM) with ANOVA and Duncan’s multiple range test were performed in SAS 9.4 (SAS Institute Inc., Raleigh, NC) to assess differences in bark anatomical traits among *Picea* species. Pearson’s correlation analysis and principal component analysis (PCA) were used to assess the relationships between different bark anatomical traits and their associations with climatic factors of their native range (Ouyang et al., 2021; Meng et al., 2022). Independent sample T-tests were used to analyze differences in bark anatomical traits under dry and moist conditions. The phylogenetic tree of this study was plotted online in ChiPlot (http://www.chiplot.online/). All statistical analysis was conducted in the R 3.6.3 software unless specified and *P* < 0.05 was considered statistically significant.

## 3 Results

### 3.1 Bark anatomical traits and their interspecific differences

**Figure 3:**
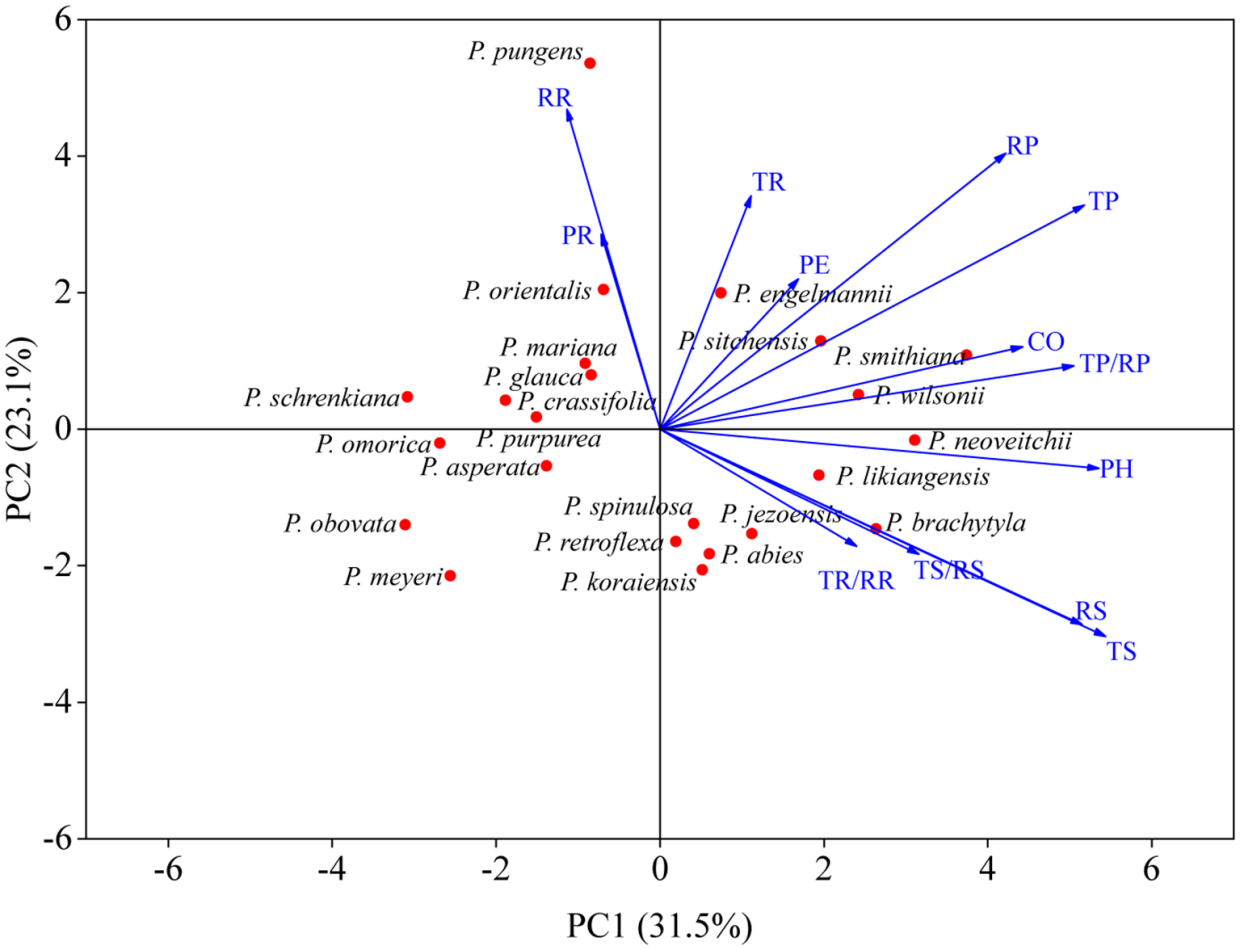
Principal component analysis of bark anatomical traits. For trait abbreviations, see **Table 1**.

The bark of the 23 *Picea* species consisted of similar tissues (**Figure 1** and **Supplementary Figure 1**). The outer bark is mainly consisted of a thick periderm and a thin rhytidome. The periderm had suberized filling tissue (A looser tissue arising outward from the phellogen in the lenticels) or phellem cells with polyphenolic content and also included unsuberized filling tissue. Between the cortex and the periderm was phellogen, with more neatly arranged cells. The cortex included resin ducts, sieve cells, and parenchyma cells, and the secondary resin ducts were mainly composed of about 2–3 layers of epithelial cells. The phloem was composed of multiple layers of parenchyma cells, sieve cells, and radially distributed phloem rays, however, the sieve cells in the secondary phloem collapsed (**Figure 1**). Among all the observed species, *P. pungens* had the largest RR (384.92 μm), while *P. sitchensis* had the largest PH (1614.60 μm). *P. obovata* had the smallest TP (32.59 μm) (**Table 3**). The resin ducts size of *Picea* species ranges from about 132.32–803.80 μm, with large variation. ANOVA and Duncan’s multiple range test showed that there were significant differences in bark anatomical traits among the 23 *Picea* species (**Tables 3**, **4**). The principal component analysis showed that the first principal component was loaded mostly by PH and CO, while the second principal component was loaded mostly by TR, RR, and PR (**Figure 3**).

**Table 3:** Differences in bark anatomical traits of 23 *Picea* species.

Species	PE	CO	PH	TR	RR	PR	TS	RS	TP	RP	TR/RR	TS/RS	TP/RP
*P. abies*	152.44 ± 20.37lmn	1,027.07 ± 240.00fg	1,129.01 ± 399.25de	572.64 ± 358.76abc	201.14 ± 122.09cdef	25.79 ± 3.77defgh	26.04 ± 6.47bc	14.02 ± 3.47bc	44.17 ± 16.28h	32.44 ± 7.48i	3.03 ± 1.51abc	1.94 ± 0.58abc	1.35 ± 0.36ghi
*P. asperata*	263.06 ± 46.23efghi	795.62 ± 104.77jkl	574.61 ± 130.82m	381.64 ± 152.04bc	169.21 ± 50.63def	27.70 ± 7.63cdefg	19.67 ± 7.79i	10.91 ± 3.99h	49.26 ± 20.29efg	32.37 ± 8.99i	2.20 ± 0.30bc	1.86 ± 0.57cdefg	1.49 ± 0.38bcdefg
*P. brachytyla*	221.34 ± 25.44hijk	1,064.76 ± 83.57ef	1,251.31 ± 172.58c	315.49 ± 133.30bc	157.77 ± 77.20ef	28.57 ± 3.78bcdef	27.99 ± 4.37a	14.23 ± 2.63abc	59.10 ± 17.41bc	37.76 ± 7.3efg	2.08 ± 0.27bc	2.04 ± 0.52ab	1.59 ± 0.49ab
*P. crassifolia*	236.24 ± 27.53ghij	891.88 ± 226.41hij	651.91 ± 168.33lm	376.33 ± 121.88bc	192.27 ± 59.52def	31.97 ± 10.31bc	18.35 ± 4.63j	11.27 ± 2.97gh	48.94 ± 25.96efgh	35.62 ± 7.79gh	1.98 ± 0.47bc	1.69 ± 0.47gh	1.39 ± 0.81efgh
*P. engelmannii*	251.80 ± 42.18fghi	1,488.89 ± 283.90b	847.13 ± 59.70hij	530.50 ± 279.24abc	332.60 ± 129.52ab	20.12 ± 4.08i	21.08 ± 4.74fgh	11.59 ± 2.48gh	60.44 ± 12.98bc	45.38 ± 8.11a	1.54 ± 0.19c	1.88 ± 0.5bcdef	1.36 ± 0.35fghi
*P. glauca*	196.84 ± 64.79jkl	900.86 ± 133.34hij	759.96 ± 312.46jk	419.34 ± 185.22bc	234.98 ± 100.05bcdef	32.73 ± 4.05b	20.25 ± 4.15ghi	12.54 ± 2.64ef	53.50 ± 14.07de	36.44 ± 6.81fg	1.78 ± 0.11bc	1.69 ± 0.53gh	1.5 ± 0.47abcde
*P. jezoensis*	265.98 ± 55.94efgh	961.60 ± 234.90gh	1,088.06 ± 88.46ef	447.10 ± 365.68bc	177.26 ± 79.34def	23.01 ± 3.03hi	25.16 ± 5.57cd	13.39 ± 2.59cde	50.21 ± 14.67efg	32.26 ± 5.91i	2.34 ± 0.74bc	1.93 ± 0.5abcd	1.56 ± 0.38ab
*P. koraiensis*	346.41 ± 102.34c	850.69 ± 106.21hijk	1,002.67 ± 134.55fg	288.76 ± 64.54c	132.32 ± 34.41f	21.52 ± 2.81hi	24.56 ± 5.64d	14.62 ± 3.02ab	48.37 ± 15.45fgh	33.08 ± 6.20i	2.21 ± 0.29bc	1.75 ± 0.52efgh	1.50 ± 0.58abcdef
*P. likiangensis*	321.68 ± 103.65cd	886.67 ± 140.90hij	1,202.14 ± 180.66cd	403.62 ± 46.10bc	183.49 ± 26.25def	29.28 ± 4.54bcde	26.26 ± 6.50bc	14.65 ± 4.31ab	57.58 ± 18.96cd	36.49 ± 6.97fg	2.23 ± 0.33bc	1.90 ± 0.59abcde	1.56 ± 0.35ab
*P. mariana*	297.31 ± 64.77def	809.71 ± 110.74ijkl	955.14 ± 161.72g	347.21 ± 94.69bc	189.30 ± 38.89def	23.97 ± 3.69fghi	16.28 ± 3k	10.72 ± 2.42h	57.64 ± 14.45cd	39.40 ± 6.4de	1.82 ± 0.23bc	1.58 ± 0.41h	1.48 ± 0.35bcdefg
*P. meyeri*	178.70 ± 43.77klm	740.11 ± 114.94l	669.91 ± 84.29klm	325.87 ± 108.37bc	186.48 ± 58.80def	24.77 ± 6.67efgh	21.49 ± 3.49efg	13.10 ± 2.99de	36.57 ± 13.77i	27.34 ± 7.63j	1.75 ± 0.16bc	1.71 ± 0.45efgh	1.32 ± 0.28hi
*P. neoveitchii*	264.50 ± 38.30efghi	1,169.97 ± 235.23cd	1,228.93 ± 238.63c	622.69 ± 370.49ab	182.80 ± 63.94def	27.94 ± 4.46cdef	26.36 ± 5.64bc	13.43 ± 2.64cde	61.60 ± 17.69bc	40.29 ± 6.51cd	3.41 ± 1.52ab	2.06 ± 0.69a	1.54 ± 0.46abc
*P. obovata*	283.13 ± 36.69defg	718.70 ± 137.45l	744.17 ± 238.56jkl	411.34 ± 190.54bc	243.82 ± 102.56bcdef	23.24 ± 2.89ghi	19.98 ± 5.26hi	11.02 ± 2.90gh	32.59 ± 9.15i	27.20 ± 6.74j	1.67 ± 0.19bc	1.88 ± 0.54bcdef	1.20 ± 0.15j
*P. omorika*	172.56 ± 28.90lmn	718.76 ± 136.24l	686.18 ± 109.54kl	383.08 ± 235.28bc	189.88 ± 148.63def	28.86 ± 5.44bcde	16.89 ± 3.77k	9.77 ± 1.93i	45.46 ± 13.60gh	34.19 ± 6.11hi	2.29 ± 0.57bc	1.77 ± 0.43cdefg	1.33 ± 0.34hi
*P. orientalis*	131.14 ± 22.10n	1,108.09 ± 111.82def	843.60 ± 165.93hij	478.49 ± 286.42bc	310.23 ± 186.20abc	29.21 ± 4.17bcde	17.12 ± 4.35jk	9.47 ± 1.99i	59.08 ± 16.02bc	39.33 ± 7.27de	1.59 ± 0.22c	1.88 ± 0.59bcdef	1.53 ± 0.45abcd
*P. pungens*	442.66 ± 159.88a	777.27 ± 191.99kl	578.32 ± 359.03m	803.80 ± 713.79a	384.92 ± 309.76a	37.09 ± 8.79a	16.32 ± 5.11k	9.78 ± 2.79i	63.28 ± 13.28b	42.10 ± 9.02bc	2.03 ± 0.25bc	1.71 ± 0.5fgh	1.54 ± 0.35abc
*P. purpurea*	301.63 ± 132.18de	865.64 ± 213.61hijk	770.77 ± 136.88jk	410.74 ± 269.33bc	254.43 ± 161.22bcde	25.46 ± 2.67defgh	19.64 ± 3.72i	11.9 ± 3.11fg	46.43 ± 15.52fgh	32.92 ± 6.86i	1.59 ± 0.17c	1.75 ± 0.56efgh	1.42 ± 0.47cdefgh
*P. retroflexa*	236.46 ± 42.00ghij	954.01 ± 148.22gh	767.71 ± 168.03jk	345.80 ± 152.93bc	175.00 ± 52.46def	29.91 ± 6.71bcd	26.42 ± 5.21bc	14.88 ± 3.45ab	47.04 ± 15.95fgh	31.99 ± 6.84i	1.93 ± 0.34bc	1.85 ± 0.46cdefg	1.47 ± 0.42bcdefg
*P. schrenkiana*	144.30 ± 36.60mn	917.04 ± 40.74hi	909.10 ± 224.13ghi	578.84 ± 200.23abc	330.76 ± 99.84ab	28.66 ± 6.53bcde	17.45 ± 3.54jk	9.75 ± 2.08i	36.42 ± 14.21i	29.18 ± 8.35j	1.74 ± 0.25bc	1.86 ± 0.51cdefg	1.24 ± 0.26ij
*P. sitchensis*	387.98 ± 149.29b	1,148.50 ± 129.62cde	1,614.60 ± 420.25a	467.30 ± 226.32bc	232.76 ± 110.42bcdef	29.49 ± 3.13bcde	22.60 ± 5.97e	13.62 ± 4.24cd	59.70 ± 13.19bc	43.02 ± 7.27b	2.00 ± 0.30bc	1.75 ± 0.55defg	1.4 ± 0.28defgh
*P. smithiana*	280.39 ± 31.87defg	1,231.73 ± 77.09c	1,435.29 ± 286.86b	450.89 ± 371.14bc	253.64 ± 170.13bcde	28.32 ± 5.85bcdef	26.75 ± 4.91b	15.10 ± 3.81a	71.39 ± 19.76a	43.85 ± 9.55ab	1.69 ± 0.23bc	1.87 ± 0.54bcdef	1.64 ± 0.36a
*P. spinulosa*	169.14 ± 26.60lmn	898.92 ± 141.71hij	827.63 ± 63.33ij	585.77 ± 636.59abc	178.06 ± 158.75def	22.68 ± 3.18hi	21.69 ± 4.18ef	12.75 ± 3.73def	50.54 ± 21.77ef	32.34 ± 9.17i	4.41 ± 6.87a	1.83 ± 0.58cdefg	1.54 ± 0.45abc
*P. wilsonii*	218.68 ± 55.96ijk	1,856.20 ± 307.08a	938.55 ± 185.62gh	599.49 ± 274.20ab	276.16 ± 155.12bcd	21.64 ± 2.15hi	24.44 ± 5.21d	14.80 ± 3.23ab	56.6 ± 13.09cd	38.14 ± 8.32def	2.93 ± 2.13abc	1.74 ± 0.57efgh	1.55 ± 0.53abc

For trait abbreviations, see **TABLE 1**. The bark anatomical traits were expressed as mean ± standard deviation. All *Picea* species were 12 years old. Unit: μm.

**Table 4:** ANOVA of bark anatomical traits among 23 *Picea* species. For trait abbreviations, see **Table 1**.

Variance	Statistics	PE	CO	PH	TR	RR	PR	TS	RS	TP	RP	TR/RR	TS/RS	TP/RP
Interspecific	F	29.56	59.01	67.39	1.91	3.68	8.31	73.29	41.19	37.16	51.13	1.74	4.66	7.33
	P	< 0.001	< 0.001	< 0.001	< 0.05	< 0.001	< 0.001	< 0.001	< 0.001	< 0.001	< 0.001	< 0.05	< 0.001	< 0.001
Intraspecific	F	7.92	8.17	16.11	2.06	3.14	3.02	19.47	11.65	11.51	11.44	1.20	4.89	5.24
	P	< 0.001	< 0.001	< 0.001	< 0.001	< 0.001	< 0.001	< 0.001	< 0.001	< 0.001	< 0.001	0.2059	< 0.001	< 0.001

### 3.2 Correlations between bark anatomical traits

**Figure 4:**
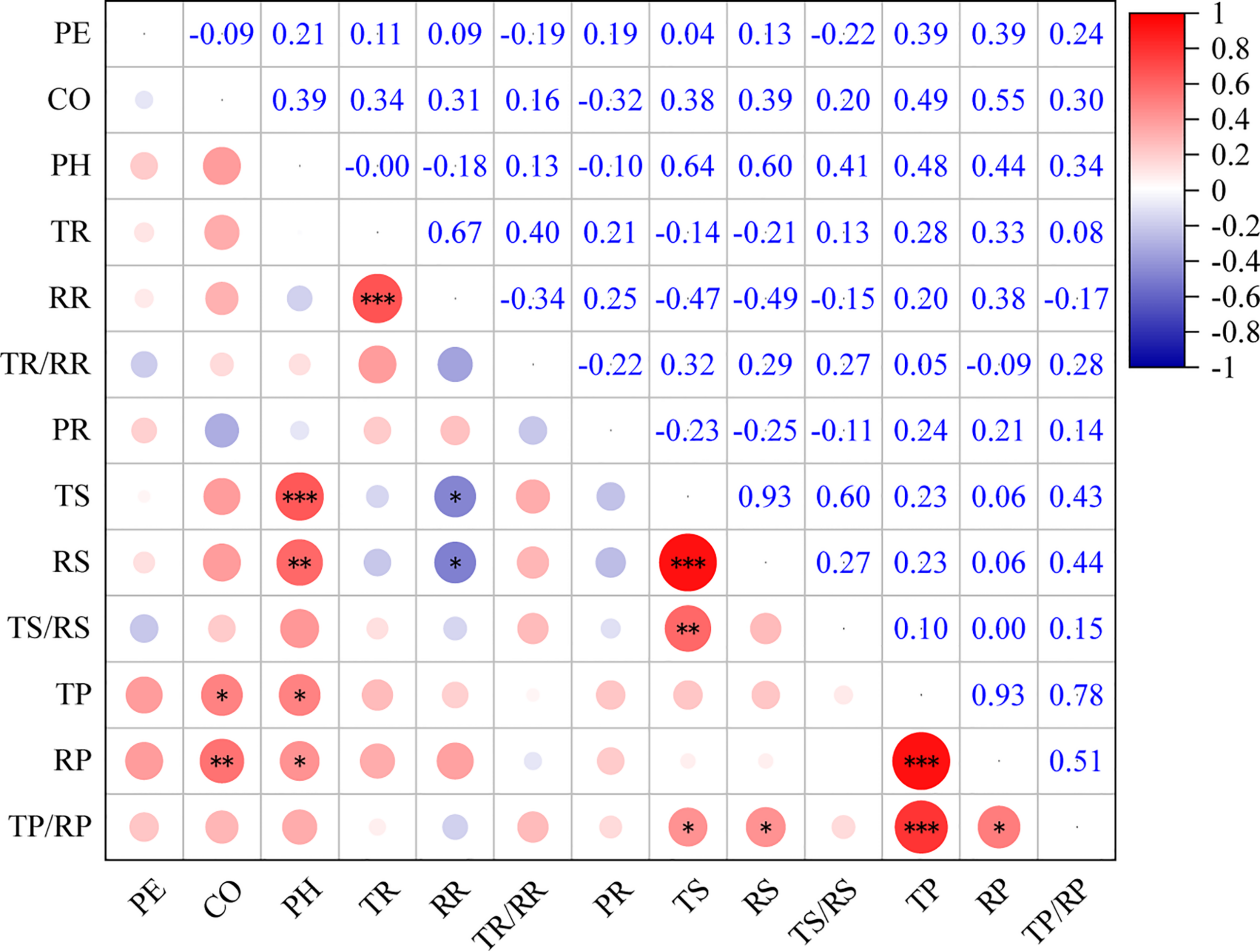
Correlations among bark anatomical traits of 23 *Picea* species. *: *P* < 0.05, **: *P* < 0.01, ***: *P* < 0.001. For trait abbreviations, see **Table 1**.

The present study showed that there were significant correlations between the bark anatomical traits of *Picea* species (**Figure 4**). There was a significant positive correlation between phloem thickness and parenchyma cell size (*P* < 0.05). There was a significant negative correlation between sieve cell size and RR (*P* < 0.05). The aspect ratio of parenchyma cells was significantly and positively correlated with sieve cell size (*P* < 0.05). There was a highly significant positive correlation between TS and PH (*P* < 0.001).

### 3.3 Phylogenetic signals of bark anatomical traits

**Figure 5:**
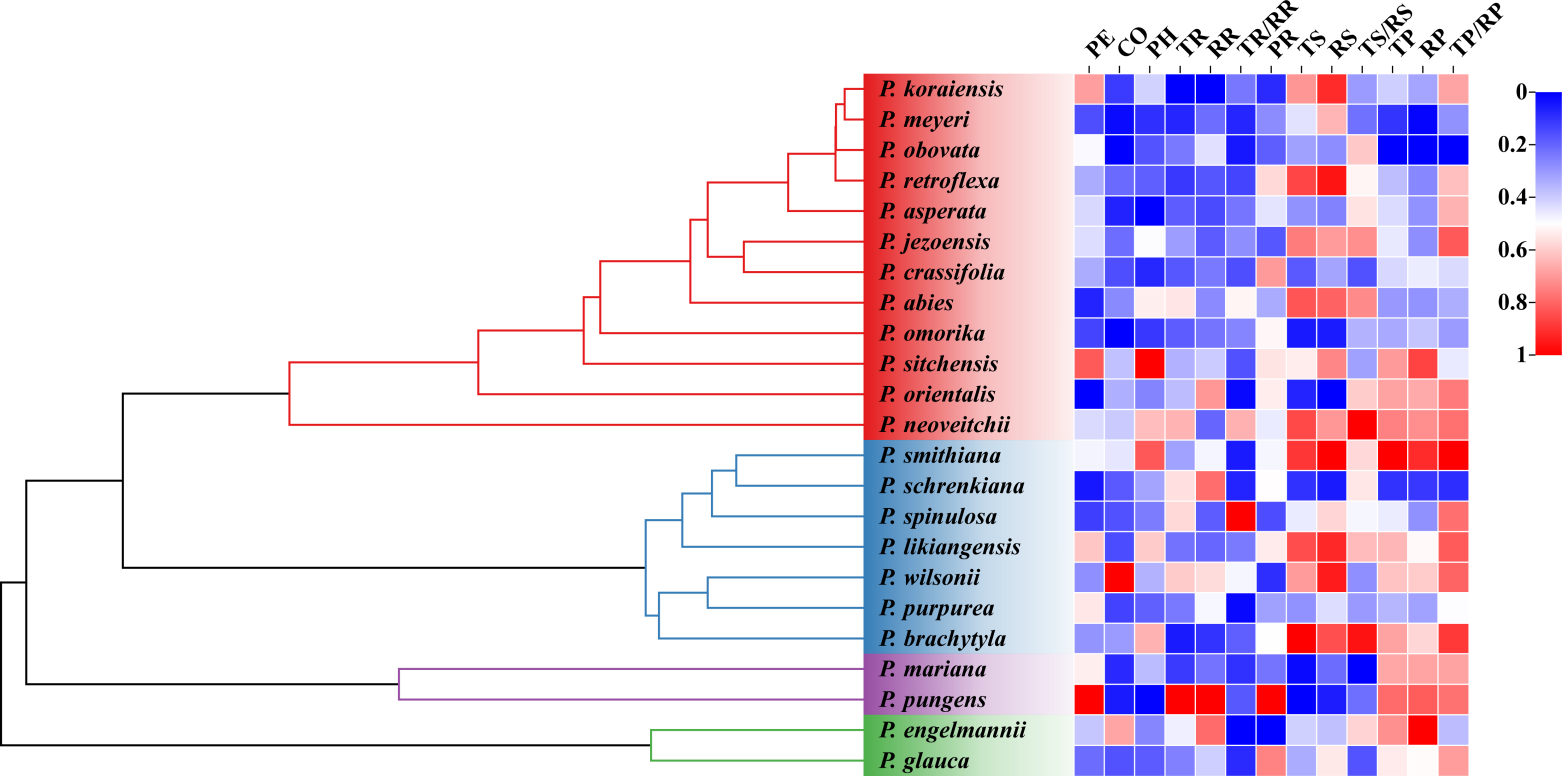
Phylogenetic relationships of *Picea* species and the variability in their bark anatomical traits. Different heatmap colors represent different bark anatomical trait sizes. All bark anatomical traits were normalized to 0-1. For trait abbreviations, see **Table 1**.

The analysis of phylogenetic signals showed that TR had a weak phylogenetic signal (Blomberg’s K = 0.47, *P* < 0.05). However, none of the other bark anatomical traits of *Picea* species had significant phylogenetic signals (**Table 5** and **Figure 5**).

**Table 5:** Phylogenetic signals of bark anatomical traits.

Bark anatomical traits	Pagel’s λ	Blomberg’s K
PE	< 0.001	0.17
CO	0.05	0.30
PH	< 0.001	0.25
TR	0.94	0.47*
RR	0.39	0.33
TR/RR	< 0.001	0.26
PR	< 0.001	0.20
TS	< 0.001	0.23
RS	< 0.001	0.19
TS/RS	< 0.001	0.27
TP	0.24	0.25
RP	0.48	0.39
TP/RP	< 0.001	0.12

*: *P* < 0.05. For trait abbreviations, see **Table 1**.

### 3.4 Relationships between bark anatomical traits and climatic factors

**Figure 6:**
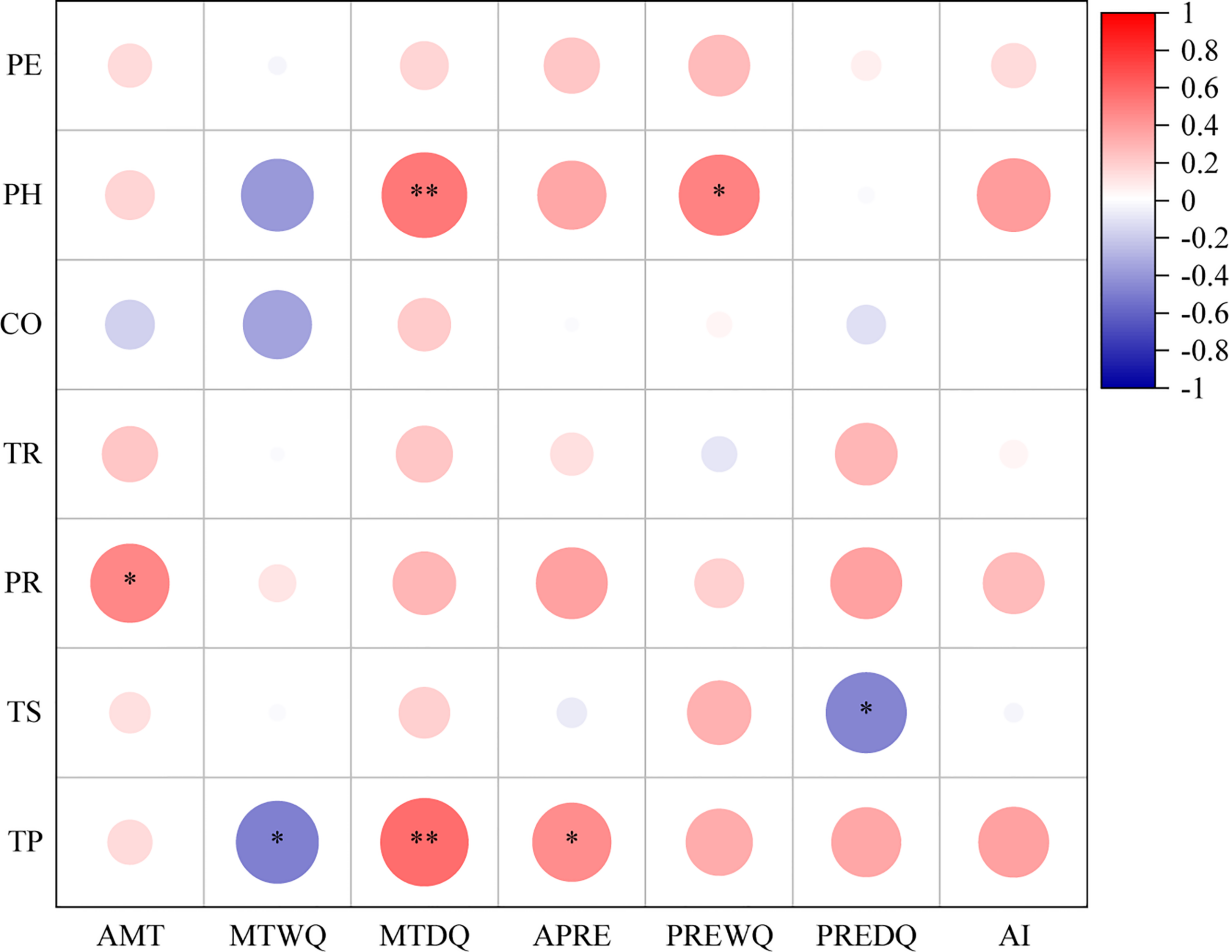
Correlation between bark anatomical traits and climatic factors in 23 *Picea* species. PE, periderm thickness; CO, cortex thickness; PHm phloem thickness; TRm tangential diameter of resin duct; PR, width of phloem ray; TS, tangential diameter of sieve cell; TP, tangential diameter of parenchyma cell; AMT, annual mean temperature; MTWQ, mean temperature of the wettest quarter; MTDQ, mean temperature of the driest quarter; APRE, annual precipitation; PREWQ, precipitation of the wettest quarter; PREDQ, precipitation of the driest quarter; AI, aridity index. All climatic factors were acquired in their native range. *: *P* < 0.05, **: *P* < 0.01.

The bark anatomical traits of *Picea* species were influenced by climatic factors of their native range (**Figure 6**). PR exhibited a positive correlation with the annual mean temperature (*P* < 0.05), while both PH and TP were positively correlated with the mean temperature of the driest quarter (*P* < 0.01). TP was positively correlated with annual precipitation (*P* < 0.05). However, there was a negative correlation between TS and precipitation of the driest quarter (*P* < 0.05). For all *Picea* species, TR was not correlated with any climatic factor (*P* > 0.05). These results clearly indicated that climatic factors of their native range had driving effects on bark anatomical traits. In addition, we found no significant differences (*P* > 0.05) in bark anatomical traits between dry and moist conditions (**Supplementary Figure 2**). This study suggested that the PH and TP of bark in *Picea* species are driven by temperature-related factors of their native range while TS is influenced by precipitation-related factors, but all traits are relatively insensitive to moist and dry environments at the global scale.

## 4 Discussion

In this study, we assessed the interspecific patterns in bark anatomical traits of *Picea* species and analyzed the phylogenetic relationships and climatic drivers underlying the differences in bark anatomical traits. The large variability in bark anatomical traits among the 23 *Picea* species suggested that species had a significant influence on bark anatomical traits. In addition, some of the bark anatomical traits exhibited synergistic relationships, there were no obvious trade-offs between traits. In general, bark anatomical traits showed only a slight influence from phylogeny, but strong influences from climatic factors of their native range.

### 4.1 Functions and interspecific variation in bark anatomical traits

It is generally accepted that tree phenotypes are shaped by a combination of genetic, developmental, and environmental factors (Coleman et al., 1994). Our study revealed specific interspecific differences in anatomical traits of the outer and inner bark, suggesting that the bark performs different functions among species. Sieve cells mainly perform the axial transport of assimilates, whereas parenchyma cells perform the radial transport of assimilates (Deng et al., 2020). Different bark anatomical structures determine bark functional traits, for example, differences in the radial structures of the phloem lead to discontinuities in the radial distribution of *in-situ* water content and saturation osmotic potential (Rosner et al., 2001). Bark is multifunctional and involved in the transport of photosynthetic assimilates, transpiration of plants, mechanical support, and defense against fire, insects, and pathogens. Bark often has lenticels that regulate water loss under dry conditions (Lendzian, 2006). Studies have shown that bark transpiration under drought conditions can be the source of more than half of the water loss from the plant and that transpiration from bark is generally a passive process not associated with plant metabolism (Lintunen et al., 2021). This water dissipation function tends to be more closely related to the outer bark (Loram-Lourenço et al., 2022), while water storage and transport are more related to the structure and function of the inner bark (Loram-Lourenço et al., 2020). *Picea* species typically have lenticels in the outer bark associated with water loss (Rosner and Kartusch, 2003), but lenticel anatomy was not studied here. Cortex contains chloroplasts that perform photosynthesis, reduce CO_2_ production by the stem, and prevent acidification of the cortex (Pfanz, 2008). Bark tissues have ecological strategies appropriate to the environment of different locations, with thicker bark occurring in fire-prone areas and thinner bark in tropical areas (Paine et al., 2010). Similarly, bark in areas with high insect infestation rates exhibited a denser composition and other induced defense strategies (Franceschi et al., 2005) associated with specific bark structures.

In addition, the structure of the phloem in the bark changes with age. For example, sieve cells tend to accumulate and take on irregular shapes. Furthermore, the thickening of the cell walls of parenchyma cells can turn them into stone cells, thus halting their cellular activity (Schweingruber et al., 2019). All species in our study were 12 years old, which controlled for any error caused by differences in age. There were significant interspecific differences in bark anatomy, which is similar to the results of interspecific differences in needle anatomy of *Picea* (Wang et al., 2021). All growth conditions in our common garden experiment were consistent, so we concluded that genetic factors had an important influence. Correlation analysis and principal component analysis of bark anatomical traits revealed significant positive correlations among most anatomical traits, correlations which were consistent even when the phylogenetic independent contrasts were resolved (**Supplementary Figure 3**). This indicated there were strong correlations among different bark anatomical traits. Both phloem thickness and parenchyma cell size were significantly and positively correlated, which may have been related to the involvement of parenchyma cells in the radial transport of assimilates. Sieve cell size was significantly negatively correlated with the radial diameter of resin ducts, and the variation of sieve cells might have been related to the expansion of resin ducts (Esau, 1969). Previous studies have shown that the swelling of the sieve cells was accompanied by a decrease in the number of resin ducts, which had a positive effect on the resistance of the plant (Krokene et al., 2008). Our results demonstrated that bark anatomical traits were generally located along the same axis in the principal component analysis, which indicated that there were no trade-offs among them, but mostly synergistic or complementary relationships. Thus, this study provides new insights into the unique position in which the bark economics spectrum (BES) is located in the plant economics spectrum (PES) (Li et al., 2022).

### 4.2 Drivers of variation in bark anatomical traits of *Picea* species

Except for TR, there were no significant phylogenetic signals for bark anatomical traits in any of the 23 *Picea* species. This indicated that phylogeny has little influence on the variation in bark anatomical traits, which was similar to the observation that bark thickness traits of angiosperms were not significantly influenced by phylogeny (Rosell et al., 2014). Our analysis indicated that climatic factors of their native range have a strong influence on bark anatomical traits. Martín-Sanz et al. (2019) found that dry conditions were not conducive to bark biomass partitioning and thus led to thinner bark, which is generally consistent with the positive correlation between the precipitation of the wettest quarter (PREWQ) and phloem thickness in our study. The mean temperature of the driest quarter had a strong effect on parenchyma cell size, which increased significantly with increasing temperature. There can also be seasonal differences in saturated osmotic pressure in the secondary phloem (Rosner et al., 2001), which may be related to the climate-driven nature of the phloem structure. Previous studies have revealed associations between parenchyma cell size and the partitioning of nonstructural carbohydrates, a mechanism of assimilate partitioning that regulates osmotic pressure in the phloem and is associated with plant resistance to embolism (Janssen et al., 2020). Poorter et al. (2014) concluded that there were no significant differences in bark characteristics between dry and moist forests, however, our results suggested that bark anatomical traits were more strongly driven by temperature than moisture conditions.

Previous studies have shown that resin ducts are associated with conifer defense functions, with wide resin ducts providing more resin and more effective protection to conifers (Mason et al., 2019). It has also been shown that the number of resin ducts is strongly correlated with temperature (Novak et al., 2013), which is consistent with our finding of a weak correlation between resin duct size and temperature. There was a weak negative correlation between resin ducts and the precipitation of the wettest quarter (PREWQ) in our study, suggesting that resin production may be reduced under less stressful conditions. Bark surface insect activity and microbial composition are often linked to bark anatomical traits. For example, the bark structure of inverted wood is associated with invertebrate and microbial composition within communities, and the greater the overall variation in bark traits, the greater the variation in faunal community composition and species richness on its surface (Zuo et al., 2016). The cortex and phloem are mostly living tissues consisting of multiple layers of parenchyma cells and sieve cells in which chemicals such as phenolics, terpenoid resins, and alkaloids provide a second layer of protection for the tree (Franceschi et al., 2005). In conclusion, bark exhibits corresponding physiological and ecological strategies when disturbed by biotic and abiotic factors.

## 5 Conclusions

Our common garden experiment revealed large interspecific differences in bark anatomical traits. Bark anatomical traits were not phylogenetically influenced but were influenced by climatic conditions of the plant origin. Interspecific variation in bark anatomical traits is an important driver of multifunctional variation in bark. It appeared that all bark anatomical traits contributed in the same dimension, influencing to the mechanical and chemical defenses of the plant. In addition, bark anatomical traits were driven by climatic factors at the seed source, which may indicate long-term climatic adaptation by the plants. Our study provided important new insight into the variability of bark anatomical traits in *Picea*, but this effort should be followed by controlled experiments to elucidate the plasticity of bark structures and use molecular techniques to reveal the functions and mechanisms of bark structural variation.

## Data availability statement

The original contributions presented in the study are included in the article/**Supplementary Material**. Further inquiries can be directed to the corresponding authors.

## Author contributions

WN contributed to execute the experiment, data analysis, manuscript drafting. YD, YL, CT, YW, and YY conducted field data collection. WX, JL, JM, and SA provided experimental guidance. ZPJ, ZRJ, and JW designed the study. All authors contributed to the article and approved the submitted version.
