# Supplementary material for: Climatic responses and variability in bark anatomical traits of 23 Picea species
Source: Front Plant Sci. 2023 Jul 17;14:1201553. doi: 10.3389/fpls.2023.1201553 (PMC10388546; doi:10.3389/fpls.2023.1201553)
Supplement: Supplementary file 1 [file DataSheet_1.docx]

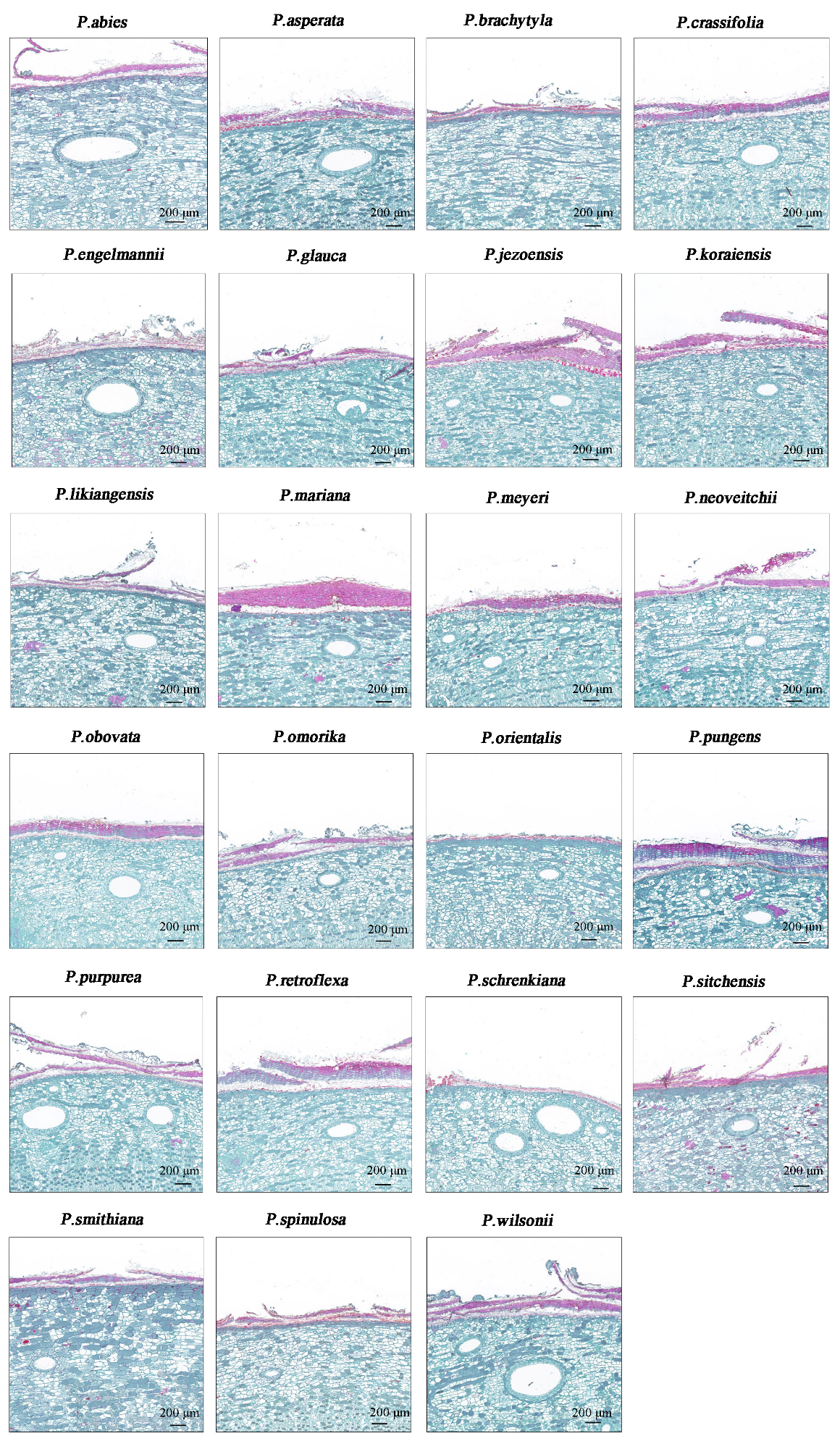


**FIGURE S1 |** Bark anatomical structures for the 23 studied *Picea* species.


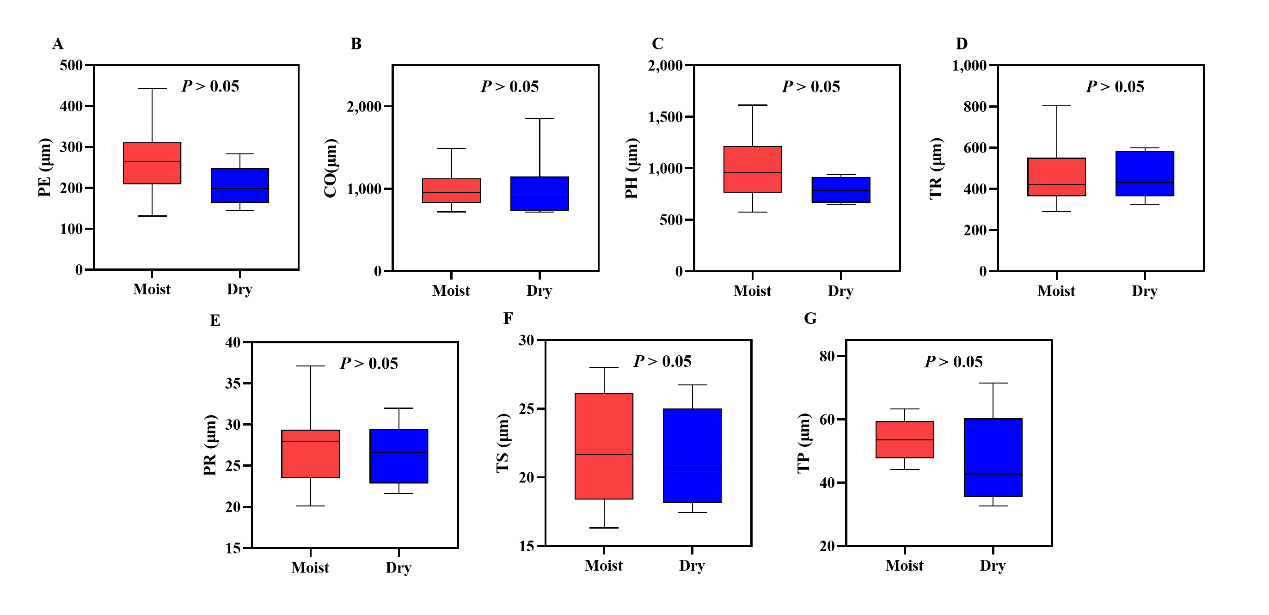


**FIGURE S2 |** Differences in bark anatomical traits of *Picea* species under different climatic conditions. For trait abbreviations, see **TABLE 1**.


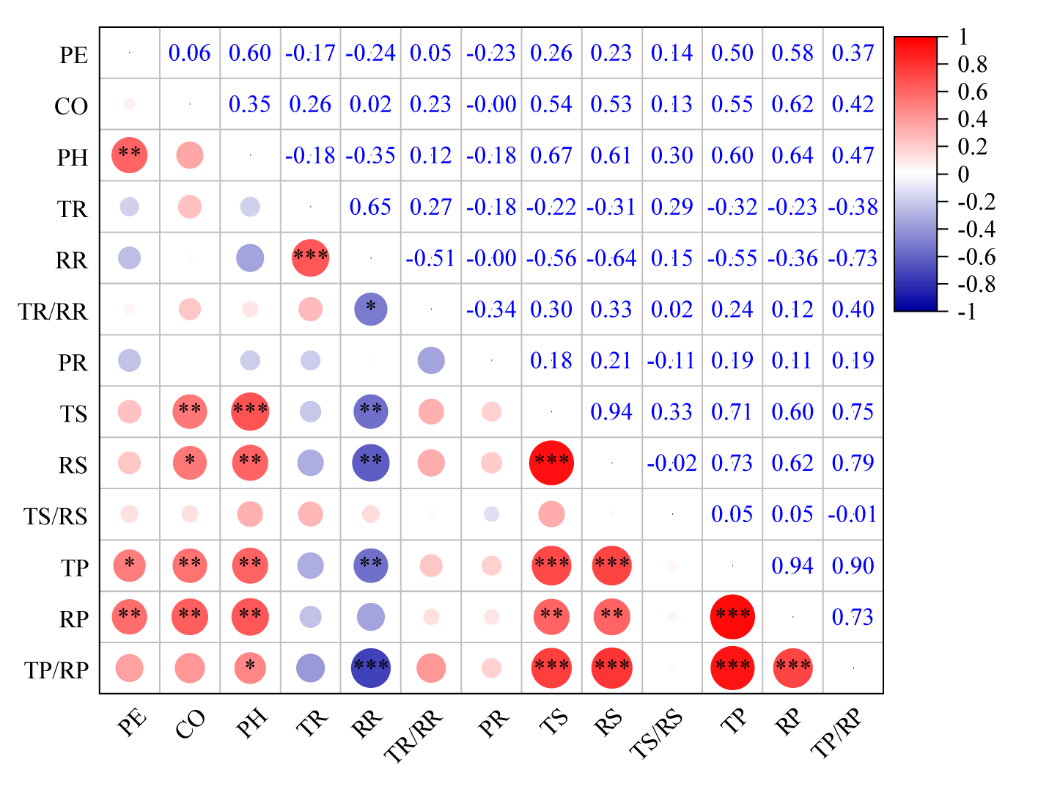


**FIGURE S3 |** Correlations among bark anatomical traits of 23 *Picea* species were considered under the condition of PIC. *: *P* < 0.05, **: *P* < 0.01, ***: *P* < 0.001. For trait abbreviations, see **TABLE 1**.
